# Anti-Virulence Properties of Curcumin/CuO-NPs and Their Role in Accelerating Wound Healing In Vivo

**DOI:** 10.3390/medicina61030515

**Published:** 2025-03-17

**Authors:** Amr M. Shehabeldine, Bahaa M. Badr, Fathy M. Elkady, Toru Watanabe, Mostafa A. Abdel-Maksoud, Abdulaziz M. Alamri, Salman Alrokayan, Amer M. Abdelaziz

**Affiliations:** 1 Botany and Microbiology Department, Faculty of Science, Al-Azhar University, Cairo 11884, Egypt; amermorsy@azhar.edu.eg; 2 Academy of Scientific Research & Technology (ASRT), 101 Kasr Al-Ainy St., Cairo 11516, Egypt; 3 Department of Basic Medical and Dental Sciences, Faculty of Dentistry, Zarqa University, Zarqa 132222, Jordan; b2badr@zu.edu.jo; 4 Department of Medical Microbiology and Immunology, Faculty of Medicine, Al-Azhar University (Assiut Branch), Assiut 71524, Egypt; 5 Microbiology and Immunology Department, Faculty of Pharmacy (Boys), Al-Azhar University, Cairo 11884, Egypt; fathyelkady2426.el@azhar.edu.eg; 6 Department of Food, Life and Environmental Sciences, Yamagata University, Yamagata 997-8555, Japan; to-ru@td1.tr.yamagata-u.ac.jp; 7 Chair of Biomedical Applications of Nanomaterials, Department of Biochemistry, College of Sciences, King Saudi University, P.O. Box 2455, Riyadh 11451, Saudi Arabia; mabdmaksud@ksu.edu.sa (M.A.A.-M.); abalamri@ksu.edu.sa (A.M.A.)

**Keywords:** CuO-NPs, curcumin, wound healing, virulence factors, *Pseudomonas aeruginosa*

## Abstract

*Background and Objectives*: This study introduces an innovative approach to accelerating wound healing by leveraging the bactericidal properties of mycosynthesized copper oxide nanoparticles (CuO-NPs) and their combination with curcumin against *Pseudomonas aeruginosa*. The study aims to evaluate their antimicrobial efficacy, impact on quorum sensing-associated virulence factors, and potential therapeutic applications in wound healing. *Materials and Methods*: The minimum inhibitory concentration (MIC) and minimum bactericidal concentration (MBC) of CuO-NPs were determined to be 25 μg/mL and 50 μg/mL, respectively. At sub-inhibitory concentrations (0.5 MIC, 0.25 MIC, and 0.125 MIC), their effects on *P. aeruginosa* growth and quorum sensing-associated virulence factors were assessed. Antioxidant activity and cytotoxicity were also evaluated. Additionally, the combination of CuO-NPs and curcumin (CUR) was tested for its enhanced wound-healing efficacy. *Results*: While CuO-NPs did not inhibit *P. aeruginosa* growth at sub-inhibitory concentrations, they significantly reduced quorum sensing-associated virulence factors in a dose-dependent manner: LasB elastase (81.8%, 60.6%, and 53.03%), LasA protease (70%, 68.5%, and 57.1%), and pyocyanin (85.7%, 71.4%, and 55.9%). CuO-NPs exhibited strong antioxidant activity by scavenging free radicals. The combination of CuO-NPs and CUR demonstrated the highest wound-healing efficacy, outperforming the negative control and Mebo ointment by 193.9% and 61.6%, respectively. Additionally, CuO-NPs exhibited selective cytotoxicity against HepG2 cancer cells while displaying minimal toxicity toward normal human skin cells. *Conclusions*: CuO-NPs, particularly in combination with CUR, show promising potential as a therapeutic agent for wound healing by inhibiting quorum sensing-associated virulence factors, exhibiting strong antioxidant activity, and demonstrating selective cytotoxicity. These findings highlight their potential biomedical applications.

## 1. Introduction

Curcumin (CUR), a bioactive compound derived from *Curcuma longa*, has been widely recognized for its therapeutic benefits, particularly in wound healing. It demonstrates antimicrobial properties by disrupting bacterial cell membranes and inhibiting virulence factor production. Additionally, its anti-inflammatory and antioxidant activities contribute to regulating different stages of the healing process by reducing oxidative stress and modulating immune responses [1]. CUR also interferes with bacterial communication systems, limiting biofilm formation and enhancing its antimicrobial effectiveness. Given these properties, combining CUR with mycosynthesized CuO-NPs presents a promising strategy for enhancing wound-healing efficiency. Prolonging the wound-healing period presents serious difficulties for both patients and healthcare providers, including increased pressure on healthcare systems by raising the cost of care and draining resources. Several reasons contribute to this problem, including contamination of wounds with pathogenic organisms. Therefore, wound care procedures must be improved by choosing the appropriate dressing, cleaning the wound, and preventing infection [2]. Therapeutic agents are essential for wound healing because they facilitate the mending and regeneration of damaged tissues. However, there are several issues associated with the creation and use of therapeutic substances in wound healing. Adverse effects such as toxicity, allergic responses, and disruption of normal wound-healing processes are possible with certain drugs. These difficulties include safety concerns and poor effectiveness [3]. CUR, a natural product considered an effective therapeutic agent, exhibits antibacterial properties against various strains of Gram-negative and Gram-positive bacterial pathogens by disrupting bacterial membranes and inhibiting the production of virulence factors [4,5]. CUR anti-inflammatory, anti-virulence, and antioxidant abilities play a major role in aiding in wound healing [6]. CUR is a viable option for wound care because of its ability to suppress virulence factors and regulate the wound-healing phases [7].

The environmentally benign and economical nature of biological approaches for synthesizing nanoparticles has attracted significant attention recently [8]. Nanoparticles can be produced sustainably and efficiently using fungi, offering advantages such as cost-effectiveness, size control, and economic viability. Fungi are readily available and easy to cultivate, making the synthesis method financially feasible. Fungal synthesis typically occurs at room temperature and neutral pH, eliminating the need for high-energy processes [9]. If the growing environment is optimized and the process controlled, fungi can regulate the form and size of nanoparticles. Customized functions and applications are made possible through the integration of biomolecules onto the surface of nanoparticles by fungal synthesis [10]. CuO-NPs have garnered significant attention due to their unique properties and potential applications. Their antibacterial, anticancer, and wound-healing qualities make CuO-NPs promising for drug delivery [11].

The rise of antimicrobial resistance has prompted investigation of alternative tactics to control microbial infections CuO-NPs, due to their strong antibacterial activity, have become particularly intriguing and promising prospects, making them useful weapons in the fight against microbial diseases. CuO-NPs have the ability to produce reactive oxygen species (ROS), including OH ^−^ and O_2_^−^, which cause oxidative stress in microbial cells, damaging DNA, lipids, and proteins [12]. CuO-NPs can interact with microbial cell membranes, compromising their structural integrity and allowing cellular contents to leak out, ultimately leading to cell death [13]. Furthermore, CuO-NPs release copper ions (Cu^2+^), which can lead to microbial cell death by interfering with essential biological functions such as protein synthesis, enzyme activity, and DNA replication [14]. To ensure the safe use of CuO-NPs in biomedical applications, their cytotoxic effects on mammalian cells must be thoroughly assessed.

Numerous diseases and the aging process are linked to oxidative stress. CuO-NPs have demonstrated encouraging antioxidant properties, making them viable options for medical interventions. CuO-NPs can scavenge and neutralize dangerous free radicals such as reactive oxygen species (ROS) and reactive nitrogen species (RNS), preventing oxidative damage to biological components. CuO-NPs can enhance the cellular antioxidant defense system by modulating the activity of antioxidant enzymes like glutathione peroxidase (GPx), catalase (CAT), and superoxide dismutase (SOD) [15]. The antioxidant properties of CuO-NPs allow them to be incorporated into skincare products, protecting the skin from environmental factors and pathogen-induced oxidative damage.

The biological process of wound healing is intricate, comprising several stages and cellular reactions. The unique characteristics and therapeutic effects of CuO-NPs have garnered much attention for their potential significance in wound healing. CuO-NPs promote angiogenesis, essential for supplying oxygen and nutrients to the wound site, by stimulating the growth of new blood vessels. They can accelerate wound closure by promoting the migration and proliferation of several wound-healing cells, including fibroblasts, keratinocytes, and endothelial cells. CuO-NPs possess antibacterial qualities, making them useful in treating or preventing wound infections, which often hinder wound healing. By regulating the inflammatory response, CuO-NPs can reduce severe inflammation and promote a healthy healing environment. When added to ointments, gels, or dressings for wounds, they enhance the healing process [16,17].

The aim of this study was to develop innovative methods that maximize the application of therapeutic drugs in wound care. By incorporating mycosynthesized CuO-NPs into therapeutic agents, we can leverage their properties to shorten wound healing duration, thereby saving medical supplies and accelerating patient recovery.

## 2. Material and Methods

The CuO-NPs were mycosynthesized from the live cell filtrate of the fungus *Penicillium chrysogenum*. The preparation processes and full characterization of CuO-NPs were described briefly in our previous publications [18]. This study used *P. aeruginosa* PAO1, which was purchased from the American Type Culture Collection.

### 2.1. MIC and MBC Determination

With a few minor adjustments, the microplate dilution method was used to obtain the MIC [19]. To find the MICs and MBCs of the CuO-NPs, *P. aeruginosa* was grown for an indefinite amount of time before being diminished with LB medium in order to achieve the appropriate bacterial cell concentration. The CuO-NPs were diluted twice (from 2−1 to 2−10) in LB broth. Next, a 5 μL solution of *P. aeruginosa* cells (standardized to 10^5^ cfu/mL) was added to each well, and the 96-well plates were incubated aerobically at 37 °C. With the use of a microplate reader, the absorbance was determined at 600 nm. The CuO-NPs concentration at the lowest dispersion at which growth was not observed was the MIC value. A total of 5 µL of the inoculum were spotted over LB agar media in the well that showed no signs of bacterial growth after a 24 h incubation period (in the MIC experiment). The plates were incubated for 16 to 24 h at 37 °C. The observed *P. aureogonisa* growth was interpreted as bacteriostatic, whereas its absence indicated bactericidal activity.

### 2.2. Estimation of Quorum Sensing (Q.S)-Controlled Virulence Factors

The LB medium was inoculated with an experimental PAO1 colony and maintained for 24 h at 37 °C with regular agitation. After collecting, the resulting solution was filter-sterilized to be utilized in the subsequent analyses.

### 2.3. LasA Protease Assay

An azocasein test was used to assess the amount of LasA protease produced in the supernatants of *P. aeruginosa* PAO1 that were either naïve or administered CuO-NPs [20]. A total of 150 μL of purified effluent and 250 μL of 2% azocasein were combined for each specimen in a 50 mM Tris-HCl buffer (pH = 7.8). This combination underwent incubation for 4h at 4 °C, when 1.2 mL of 10% trichloroacetic acid was added to stop the reaction. The combination was then incubated for 15 min at 4 °C and centrifuged for 10 min at 10,000 rpm. Finally, the supernatant was mixed with 1.4 mL of 1 M NaOH, and the percentage of protease activity was measured as the OD440 of the supernatant.

### 2.4. LasB Elastase Assay

In brief, 1 mL of produced resultant filtration was subjected to incubation at 37 °C for 16 h with shaking with 1 mL of elastin Congo red (ECR), which consists of (10 mg/mL in 100 mM Tris-HCl, pH = 7.5; 1 mM CaCl_2_) reaction buffer. To eliminate residual ECR, the mixture was centrifuged at 3000× *g* for 10 min, and the OD495 was used to measure elastase activity.

### 2.5. Pyocyanin Assay

The pyocyanin content was determined by mixing 7.5 mL of purified effluent with 4.5 mL of chloroform and stirring till the color turned to a greenish blue. The specimens were centrifuged (10,000× *g* for 10 min), and 3 mL of the resultant blue fluid was moved to a new tube holding 1.5 mL of 0.2 M HCl and stirred till the blue color became pink. The pink-colored surface was put on to a cuvette, and its absorption was measured using the OD520.

### 2.6. Antioxidant Property of CuO-NPs

A total of 100 μL of CuO-NPs and 0.5 mL of 0.1 mM DPPH solution were prepared using 95% ethanol, the final approach maintained in secrecy. Ten distinct levels of the tested particulate solution were used and 50% MeOH and butylated hydroxytoluene (BHT) were regarded as positive and negative controls, respectively. To ascertain the IC_50_ of CuO-NPs (IC50 is the quantity necessary to disable 50% of the antioxidant effect), after that, the absorbance of the specimens was measured at 518 nm [21]. The following formula was used to determine the percentage of radicalization activity.

Inhibition% = Sample A/ControlA × 100%

### 2.7. Cytotoxicity and Wound Healing Assay

The study’s objective was to evaluate cell survival to calculate the IC50 (half maximal inhibitory concentration) of the newly developed formulations. The cytotoxicity of these formulations was assessed on the human skin cell line (BJ-1) using the SRB assay [22]. A cell suspension containing 5 × 10^3^ cells in 100 µL was cultured in standard medium for 24 h in a 96-well plate. Subsequently, the cells were exposed to 100 µL of medium containing different concentrations of the solubilized drug formulations (CuO-NPs); Dox was used as a positive control. After a 3-day exposure, the cells were fixed by incubating for 60 min at 4 °C. Subsequently, 150 µL of 10% trichloroacetic acid was added to the media. After the TCA solution was removed, the cells underwent five rounds of washing with distilled water. A 70 µL aliquot of 0.4% *w*/*v* solution of SRB was added, and the fusion was left in the dark at 25 °C for 10 min. After 24 h drying by air, the plates were washed with 1% CH_3_COOH for 3 times. To dissolve the protein-bound SRB dye, 150 µL of TRIS (10 mM) was applied. At 540 nm, the absorbance was measured. The concentration at which 100% of the cells in each sample were viable was determined by calculating the Effective Safe Concentration (EC100) value using GraphPad Prism software (version 6.01). The cytotoxicity rate (*CT*%) was calculated using the following formula:

$$CT\%=\frac{AC-AT\;}{AC}\times 100\%$$

where *AC* and *AT* represent the absorbance of the control and test sample, respectively.

### 2.8. In Vivo Wound Healing Evaluation

All experiments adhered to the Animal Welfare Act and Rules and the Handbook for the Care and Utilization of Animals for Research Use (8th ed., Washington, DC, USA), with approval from the Animal Ethics Committee of Al-Azhar University, using Sprague Dawley rats (2 months old, 200–220 g), which were randomly assigned to four groups (Group I: untreated control, Group II: treated with Mebo ointment, (0.25% *w*/*w* ß-sitosterol) as a positive control, Group III: received CuO-NPs embedded with CUR at the MIC concentration, and Group IV: treated with 2% CUR), and after they were anesthetized with 4% pentobarbital sodium (1 mL/kg), a 10 mm circular wound was created on both sides of the backbone using a biopsy punch, followed by wound monitoring through photographs taken on days 0, 2, 4, 8, and 16, with wound area quantification performed using ImageJ software (version 1.53).

The following formula was used to determine the proportion of wound coalescence (%) (PWC)

$$\mathrm{PWC}\;(\%)=\frac{A_{0}-A_{t}}{A_{0}}\times 100\%$$

where A_0_ represents the initial wound area, and A_t_ represents the wound area at a given time point.

### 2.9. Histopathological Analysis

The extensive histological alterations at the injury area were assessed using H&E staining. Following the post-operative duration, circle specimens of scar marks were taken from the animals using an identical punch (1.5 cm diameter) after 4, 8, and 16 days, which included superficial, beneath the skin, and muscle tissues engaging membranes. The resulting tissues (samples) were preserved in a 10% neutral buffer formalin solution for around three days before being paraffin-embedded. The paraffin blocks were cut into 8 µm slices and stained with hematoxylin and eosin, as per the histological protocol. The injured locations were removed, formalin-fixed, and subsequently treated and encased in paraffin. Thick slices (3–5 µm) were stained with hematoxylin and eosin, then imaged at 200× resolution.

### 2.10. Result Statistical Analysis

All the experiments were conducted in triplicate, and the data were expressed as mean ± standard deviation (SD). Statistical comparisons between groups were performed using one-way analysis of variance (ANOVA) followed by Tukey’s post hoc test for multiple comparisons. Differences were considered statistically significant at *p* < 0.05. GraphPad Prism (version 9) was used for data visualization and statistical calculations.

## 3. Results and Discussion

### 3.1. Determination of MIC and MBC

**Figure 1:**
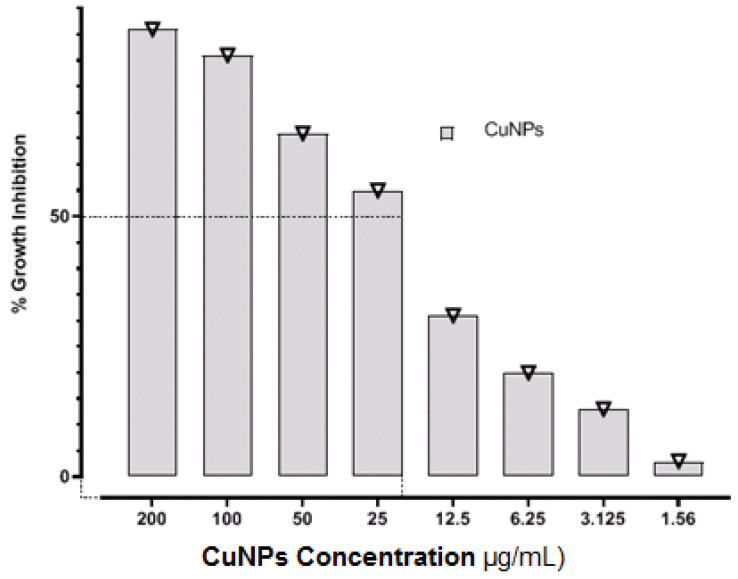
Determination of MIC of CuO-NPs against *P. aeruginosa* PAO1.

*P. aeruginosa* PAO1 at a concentration of 10^8^ CFU/mL was sensitive to CuO-NPs, as presented in Table 1 and Figure 1. The MIC was 25 μg/mL, whereas the MBC was 50 μg/mL. The antimicrobial activity of CuO-NPs has different mechanisms, including cell surface damage, ROS production, oxidative malfunction, peroxidation of fatty acids, and oxidative damage to proteins [23,24]. Some potential antibacterial processes include the contact and breakdown of the microbial cell barrier, allowing for the release of intracellular material [25,26]. There are no publications on the process of the antibacterial impact of CuO-NPs. CuO-NPs may have a similar mode of action as silver nanoparticles. The increased sensitivity of *P. aeruginosa* to copper nanoparticles may be related to a greater number of amines and carboxyl groups on the outermost layer of the bacteria, as well as a greater attraction of copper for these molecules [27,28]. Copper ions produced later may bond with DNA atoms, causing helical structural disruption via cross-linking inside and among DNA threads. Copper ions within cells of bacteria also interfere with metabolic activities [29,30].

### 3.2. Effects of CuO-NPs on Q.S-Controlled Pseudomonas aeruginosa Virulence

**Figure 2:**
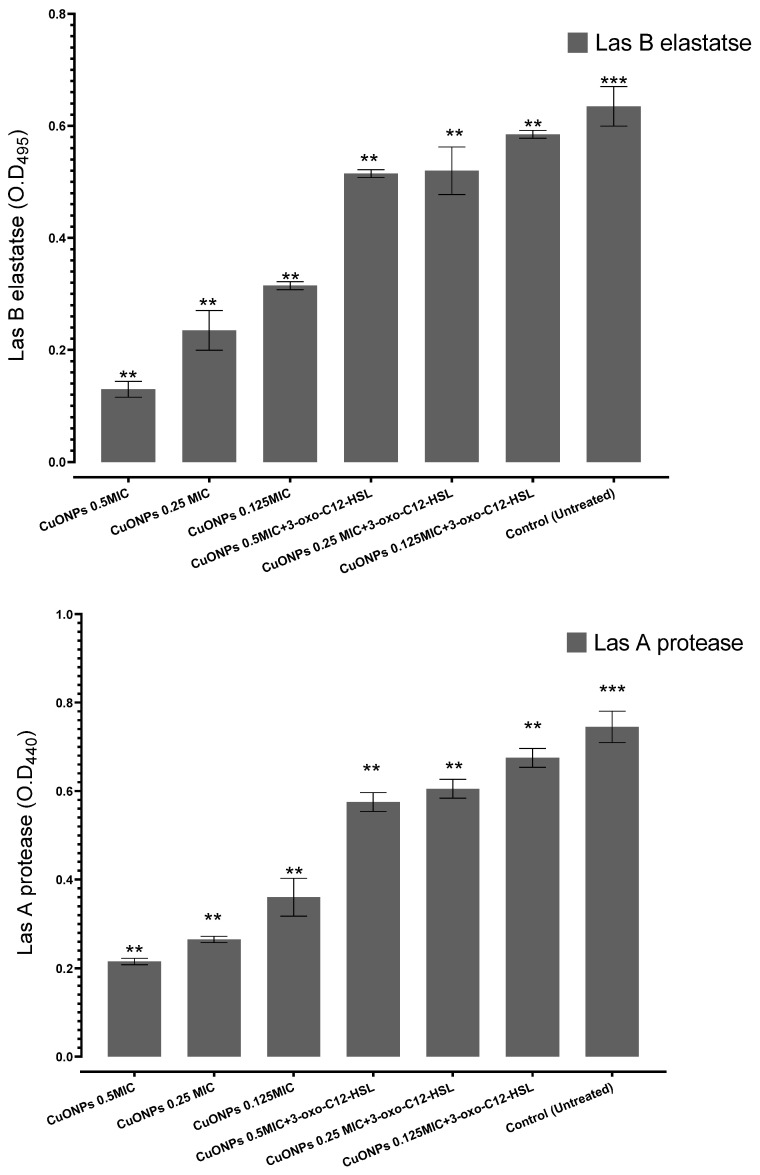

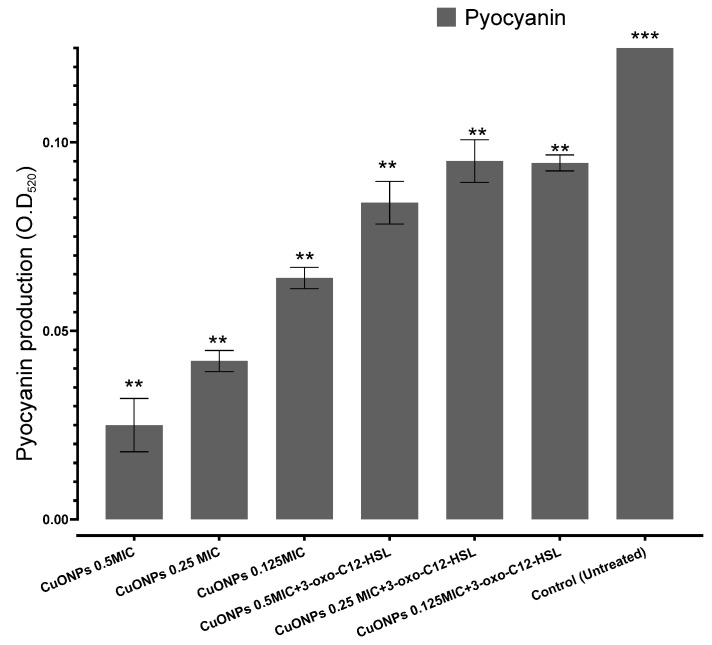
The inhibitory impact of sub-MICs of CuO-NPs on the synthesis of Q.S-regulated external virulence-related factors of *Pseudomonas aeruginosa* PAO1 culture. The standard deviations of three measurements are shown by error bars, ** *p* < 0.01, and *** *p* < 0.001 compared to the untreated group.

This study tried a novel approach to specifically interfere with Q.S mechanisms aimed at neutralizing aggressive invasive microorganisms like *P. aeruginosa* PAO1. To precisely attack Gram-negative bacteria, Q.S is often controlled by acyl-HSLs. In this work, we examined the anti-Q.S capabilities of CuO-NPs that have been shown to reduce virulence in *P. aeruginosa*. The application of Q.S inhibitor at sub-inhibitory doses (0.5–0.25 MIC and 0.125 MIC) did not affect the development profile of *P. aeruginosa* PAO1 (Figure 2). This aligns with previous reports where metallic nanoparticles, such as silver and zinc oxide nanoparticles, exhibited Q.S inhibitory effects, leading to reduced virulence in *P. aeruginosa* PAO1 [31]. To investigate the impact of CuO-NPs on the Q.S apparatus of *P. aeruginosa*, three Q.S-related virulence indicators, LasA protease, LasB elastase, and pyocyanin production, were assessed at sub-MICs (0.5 MIC–0.25 MIC and 0.125 MIC) of CuO-NPs, which dramatically decreased the production of these three Q.S-relevant virulence factors in a dose-dependent manner: 81.8, 60.6, and 53.03% suppression for LasB elastase. Similarly, our results are consistent with studies where plant-derived Q.S inhibitors, such as CUR and eugenol, demonstrated comparable suppression of LasB elastase production [32]. LasA protease was also significantly reduced by sub-MICs (0.5–0.25 MIC and 0.125 MIC) of CuO-NPs in a dose-dependent manner: 70, 68.5%, and 57.1% for LasA protease; 85.7%, 71.4%, and 55.9% suppression for pyocyanin production. Furthermore, our observed inhibition of LasA protease (70%, 68.5%, and 57.1%) is in line with the findings from [33], who reported that biofabricated nanoparticles significantly attenuated LasA protease synthesis in *P. aeruginosa*, thereby weakening its invasive potential. During the initial developmental cycles, *P. aeruginosa* PAO1 was treated with artificial 3-oxo-C_12_-HSL (0.13 M Sigma-Aldrich, St. Louis, MO, USA), and the maximal autoinducer levels of *P. aeruginosa* PAO1 were considered, to investigate if the adverse effects of CuO-NPs could be recovered by supplementing with exogenous acyl-homoserine lactones. Unexpectedly, the addition of synthetic 3-oxo-C_12_-HSL reduced the inhibitory effect of CuO-NPs on the ejection of each of the previously recognized exogenous variables in a dose-dependent manner: 22.7%, 18.3%, and 3.3% suppression for LasB elastase, while LasA protease was also significantly reduced by sub-MICs (0.5–0.25 MIC and 0.125 MIC) of CuO-NPs in a dose-dependent manner: 39, 28%, and 21.5% for LasA protease; 42.8%, 29.2%, and 31.3% suppression for pyocyanin production.

In the present investigation, we looked at the capacity of CuO-NPs at sub-MIC levels to suppress Q.S, signaling compounds, and related pathogenicity variables in virulent *P. aeruginosa*, as well as whether CuO-NPs had anti-quorum sensing action. External virulence determinants produced by *P. aeruginosa* PAO1 are considered biomarkers of the Q.S regular optimum function [34,35,36]. Their reduced manufacturing confirms the investigated test compound’s anti-Q.S potential. LasA protease and LasB elastase play essential functions in the development of *P. aeruginosa*-induced pneumonia in the lungs [37]. Significantly, the diminution of *P. aeruginosa* infectiousness by CuO-NPs could be partly due to the minimized manufacturing of HSLs, which are essential signaling molecules that stimulate the Q.S circuitry and eventually generate extracellular virulence factors. Exogenous supplemental intake with HSLs may potentially alleviate the detrimental effect of CuO-NPs on the production of external infectiousness factors.

### 3.3. Antioxidant Activity of CuO-NPs

**Figure 3:**
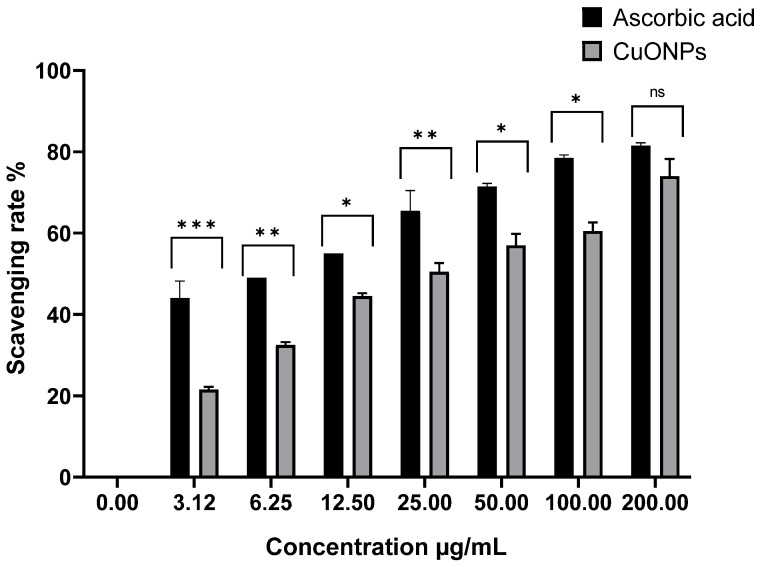
Percentage of DPPH scavenging activity at different concentrations of CuO-NPs. * *p* < 0.05, ** *p* < 0.01, and *** *p* < 0.001 compared to the positive control. “ns” (not significant) compared to the untreated group.

For the purpose of figuring out if the contact with CuO-NPs triggered an oxidative stress signal in microbes, the production of ROS was monitored. 2,2-Diphenyl-1-Picrylhydrazyl (DPPH) constitutes one of the chemicals that exhibits typical proton radical generation and integration, whose absorption decreases significantly when exposed to proton reactive gatherers [38]. Figure 2 shows the capacity of CuO-NPs and ascorbic acid to scavenge free radicals caused by DPPH. The scavenging effects increased with CuO-NPs concentration, reaching a high of 77% at 200 µg/mL (Figure 3). The ascorbic acid shielding effects ranged from 41% to 81% depending on the quantity, but no significant variations in DPPH scavenging activities were found (*p* > 0.05). Our findings align with previous studies reporting high antioxidant activity of CuO-NPs. Harishchandra et al. [39] demonstrated that CuO-NPs possess strong free radical scavenging abilities, supporting their role as effective antioxidants. Additionally, [40] found that CuO-NPs enhanced the activity of key antioxidant enzymes such as superoxide dismutase (SOD) and catalase (CAT) while reducing ROS levels in the testes of mice, reinforcing their potential in biological applications. The scavenging ability of ascorbic acid, an antioxidant, and CuO-NPs against free radicals produced by DPPH is shown in Figure 2. Based on the data, it was observed that the scavenging effects increased with the concentration of CuO-NPs, peaking at 77% at 200 µg/mL (Figure 3). These results indicated that CuO-NPs possess strong antioxidant properties, through scavenging DPPH-generated free radicals. The results showed that the scavenging capacities of CuO-NPs and ascorbic acid were quite consistent and did not show dose-dependent effects within the measured concentration range. This high antioxidant activity of CuO-NPs has been recorded by many researchers [41]. At animal levels, CuO-NPs increased the activities of SOD and CAT while lowering the amount of ROS in the testes of rats [42]. The application of CuO-NPs at the plant level involves several mechanisms as antioxidant agents, leading to a significant increase in the total phenolic content of both healthy and *F. oxysporum*-infected plants. The use of CuO-NPs resulted in decreased production of malondialdehyde (MDA) and hydrogen peroxide (H_2_O_2_) by enhancing the presence of antioxidant compounds that efficiently eliminate ROS and protect cellular membranes. Additionally, spraying with CuO-NPs enhanced the activities of peroxidase and polyphenol oxidase [43].

Possible mechanisms of action for the CuO-NPs include the electrostatic interaction that harms cell membranes, the disruption of proteins and enzymes, the generation of ROS and subsequent oxidative stress, the binding of proteins that disrupts cellular homeostasis (including the electron transport chain), the inhibition of signal transduction, and the potential genotoxicity [44].

### 3.4. Cell Migration Assay

**Figure 4:**
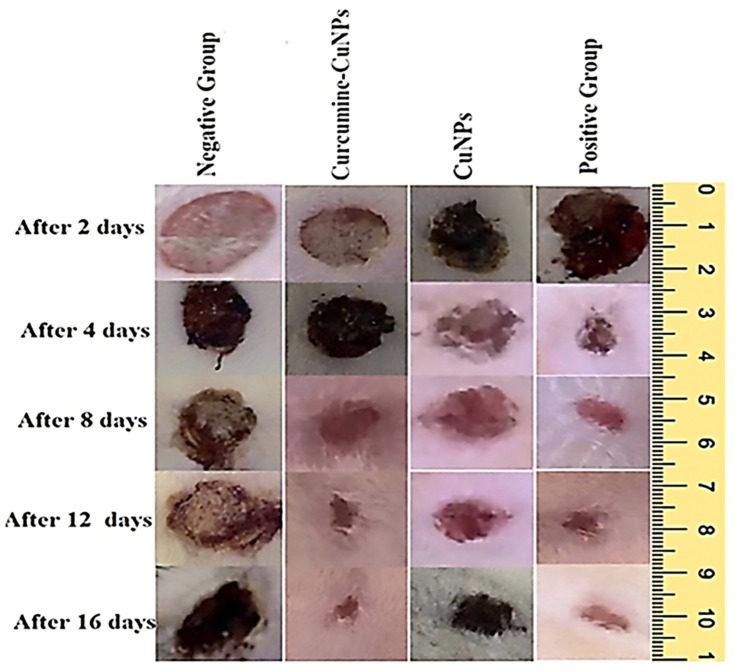
Wound closure effect of CuO-NPs and CuO-NPs-CUR on the wound-healing process compared to the untreated group.

The capacity of CuO-NPs to stimulate fibroblast migration was assayed using sub-IC_50_ concentrations applied to human skin fibroblasts. The findings demonstrated that the CuO-NPs had regeneration and wound-healing activities (Figure 4). To regenerate and repair damaged tissue following an injury, a series of wound-healing processes, including hemostasis, inflammation, proliferation, and maturation, must be involved [45]. Following the same period, treatment with CuO-NPs shows that 77% of the wound has healed, providing an improvement of 102.6% over the negative control and 28.3% more than the positive control (Mebo ointment). Turmeric contains CUR, a natural substance with anti-inflammatory and wound-healing properties. The CuO-NPs-CUR therapy was shown to significantly speed up wound healing by 193.9% compared to the negative control and 61.6% compared to the positive control. Our findings indicated that the CuO-NPs possessed significant healing activities through enhanced bactericidal and antioxidant activities. These results are explained by studies that have proven the pharmacological, bactericidal, fungicidal, and antiviral activity of CuO-NPs [17]. CUR possesses significant anti-inflammatory and antioxidant properties that can balance ROS production and antioxidant activity. It may accelerate healing by reducing the duration of the inflammatory phase, as it induces apoptosis in inflammatory cells during the early stages of wound healing. Furthermore, CUR may promote fibroblast migration, differentiation, and collagen synthesis [46].

### 3.5. Effect of Topical Application of CuO-NPs-CUR on Wound Maturity

**Figure 5:**
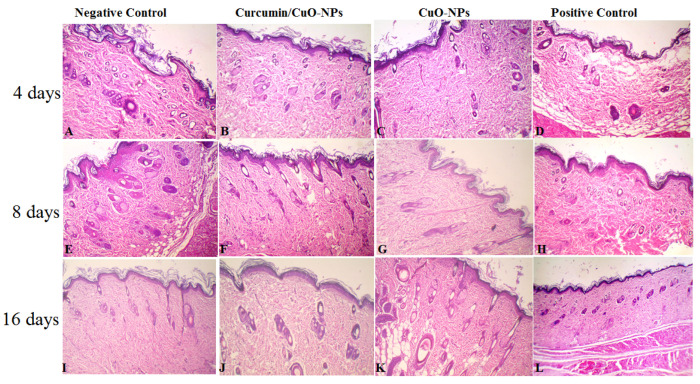
Diagrams illustrating typical histological examinations of granulation and healing regions in rats subjected to different treatments. The groups include control (**A**,**E**,**I**), CuO–NPs (**C**,**G**,**K**), CUR/CuO-NPs (**B**,**F**,**J**), and positive control (Mebo ointment, 0.25% *w*/*w* ß-sitosterol) (**D**,**H**,**L**), assessed on days 4, 8, and 16 post-wounding.

The CuO-NPs and CuO-NPs-CUR were evaluated on days 4, 8, and 16 after injury for histopathological examination. In addition, the CuO-NPs-CUR-treated category had more fibroblasts and blood vessels than the other sections (Figure 5A–D). On day 8, the CuO-NPs-treated category’s lesions showed signs of inflammation within the outermost portion and lymphocytes in the lower area (Figure 5G). Skin lesions administered with CuO-NPs-CUR showed vigorous heavy granulation tissues filled by increased circulation and fibroblasts as well as modest collagen buildup (Figure 5F). On day 16, the nonintervention category’s incised segments still showed an increased number of pro-inflammatory cells, blood vessels, and astrocytes. The untreated group’s wound sections, however, showed no signs of thick drainage formation (Figure 5I). The injured portions of the CuO-NPs-treated cohort showed a high number of fibroblast producing the protein collagen; however, the resulting collagen accumulation was not homogeneous (Figure 5K). Wounded slices from the CuO-NPs-CUR group exhibited a dense network composed of cells covered in a thick epidermal layer (Figure 5J). The histopathological score shows lesion progression. The CUR-treated group scored much higher on days 4 and 8 than the other groups. Expanding fibroblast with the production of the extracellular matrix and blood vessels oriented perpendicularly are characteristics of exceptional granulated mucosa. Mature healed wound tissue requires not only the synthesis of extracellular matrix during healing but also its ongoing breakdown and remodeling in a controlled way. Enhanced fibroblast development in injuries administered with CuO-NPs-CUR is indicative of quicker epidermal surface replacement in these wounds when compared to the other groups, which eventually resulted in faster wound closure [43]. This is consistent with a previous observation that the administration of CUR leads to an increase in the amount of collagen in the injury bed, early re-epithelialization of the epidermis, and faster wound repair [47]. Regeneration, which is hampered by diabetes, aids in closure of wounds by converting keratin cells from a stationary to a migrating and proliferating morphology [48].

### 3.6. Cytotoxic Activity

**Figure 6:**
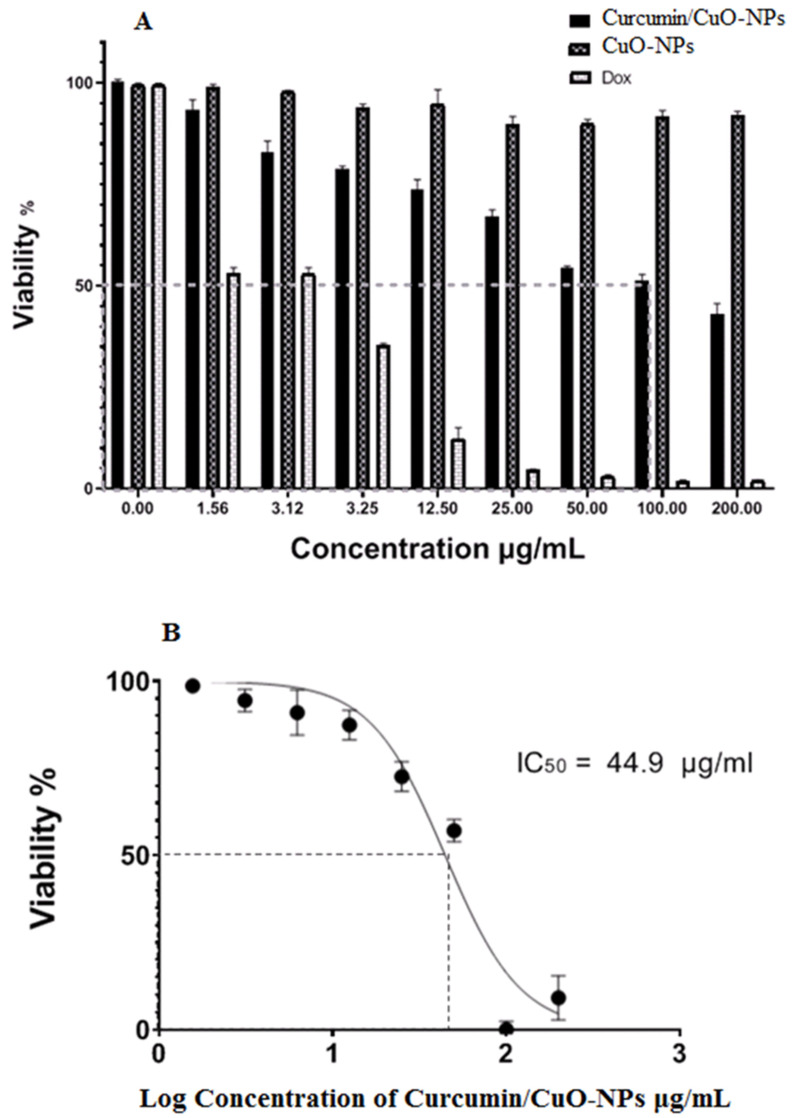
(**A**) Cytotoxic effect of CUR/CuO-NPs on the normal cell line (BJ-1). (**B**) IC50 of CUR/CuO-NPs against HepG2 cells, with Doxorubicin (Dox) used as a reference control. Data are presented as mean ± SD from three independent experiments.

Figure 6 demonstrates that there were no cytotoxic effects of the CuO-NPs on BJ-1 cells at an IC_50_ value of 100 μg/mL, while the CuO-NPs decreased HepG2 cell viability from 96.3% to 41% at concentrations ranging from 1.56 μg/mL to 200 μg/mL. However, no cytotoxic effects were observed on BJ-1 cells even at the highest concentration of CuO-NPs. Clinically significant is the preferential cytotoxicity of CuO-NPs to cancer cells relative to normal cells, indicating that these CuO-NPs possess safe medical properties [49]. CuO-NPs have been demonstrated as ROS producers, which may be involved in the primary mechanism for destroying cancer cells by damaging biological structures and inducing apoptosis [50]. In the second mechanism, CuO-NPs can modify the viability and function of the mitochondrial membrane [51]. The third pathway of CuO-NPs involves stimulating reduced glutathione, thus eliminating oxidants such as myeloperoxidase and lactoperoxidase [52].

## 4. Limitations

This study has certain limitations that should be considered. One key limitation is the need for in vivo validation to confirm the findings under physiological conditions. Additionally, bacterial responses to CuO-NPs may vary, and environmental factors could influence the observed effects. Further research is necessary to fully elucidate the underlying molecular mechanisms and assess the long-term safety of CuO-NPs for clinical application.

## 5. Conclusions

The results indicated that CuO-NPs possess a range of intriguing biological activities, including antibacterial, antioxidant, and anticancer effects. The CuO-NPs-CUR-treated group performed significantly better than the other groups. Remarkable granulation mucosa is characterized by growing fibroblasts, matrix extracellular synthesis, and parallel blood vessel orientation. These results provide insightful information for developing novel approaches to combat infections, promote wound healing, and potentially treat cancer.

## Figures and Tables

**Table 1:** Susceptibility of *P. aeruginosa* PAO1 to antimicrobial agents.

Antimicrobial Agents	MIC	MBC	MBC/MIC Ratio
(CuO-NPs) (µg/mL)	25	50	2
Levofloxacin (µg/mL)	3.25	6.5	2
Ceftazidime (µg/mL)	6.5	13	2

## Data Availability

Data are contained within the article.
